# *Strongyloides* spp. and Cytomegalovirus Co-Infection in Patient Affected by Non-Hodgkin Lymphoma

**DOI:** 10.3390/tropicalmed8060331

**Published:** 2023-06-20

**Authors:** Tommaso Lupia, Elena Crisà, Alberto Gaviraghi, Barbara Rizzello, Alessia Di Vincenzo, Fabrizio Carnevale-Schianca, Daniela Caravelli, Marco Fizzotti, Francesco Tolomeo, Umberto Vitolo, Ilaria De Benedetto, Nour Shbaklo, Alessandro Cerutti, Piero Fenu, Vanesa Gregorc, Silvia Corcione, Valeria Ghisetti, Francesco Giuseppe De Rosa

**Affiliations:** 1 Unit of Infectious Diseases, Cardinal Massaia, 14100 Asti, Italy; 2 Unit of Oncology and Haematology, Candiolo Cancer Institute, 10060 Candiolo, Italy; 3 Department of Medical Sciences, Infectious Diseases, University of Turin, 10126 Turin, Italy; 4 Microbiology Unit, Amedeo di Savoia Hospital, 10100 Turin, Italy; 5 Intensive Care Unit, IRCCS Candiolo, 10100 Candiolo, Italy; 6 Healthcare Management, IRCCS Candiolo, 10100 Candiolo, Italy; 7 School of Medicine, Tufts University, Boston, MA 02111, USA

**Keywords:** *Strongyloides*, cytomegalovirus, immunocompromised host, solid organ transplant, hematopoietic stem cell transplantation, HIV

## Abstract

To our knowledge, we have described the first case of *Strongyloides*/Cytomegalovirus (CMV) concomitant infection that occurred in a European country. The patient was a 76-year-old woman affected by relapsed non-Hodgkin lymphoma who presented interstitial pneumonia with a rapidly progressive worsening of respiratory insufficiency, leading to cardiac dysfunction and consequent death. CMV reactivation is a common complication in immunocompromised patients, while hyperinfection/disseminated strongyloidiasis (HS/DS) is rare in low endemic regions, but has been widely described in Southeast Asia and American countries. HS and DS are two consequences of the failure of infection control by the immune system: HS is the uncontrolled replication of the parasite within the host and DS the spreading of the L3 larvae in organs other than the usual replication sites. Only a few cases of HS/CMV infection have been reported in the literature, and only in one patient with lymphoma as an underlying disease. The clinical manifestations of these two infections overlap, usually leading to a delayed diagnosis and a consequent poor outcome.

## 1. Introduction

Strongyloidiasis is a parasitic infection primarily caused by the nematode *Strongyloides stercoralis* [1]. It is widely endemic in tropical and subtropical areas but is also present in temperate areas and accounts for 30–100,000 million infections worldwide [2,3]. Transmission occurs through contact between the bare skin and the filariform larvae of *Strongyloides* in the soil. The initial sign of the acute infection is localized and pruritic erythema at the skin penetration site. The rash may migrate following the movements of the larva in the derma: this phenomenon takes the name of larva currens.

From the skin filariform (L3), larvae migrate to the small intestine mucosa. There are multiple routes in which they could reach the gut; the most common is passing through the lungs and being swallowed as they ascend the airways [3]. In the intestine, L3 larvae mature in the adult female nematode, which produces eggs via parthenogenesis from which new L3 larvae hatch. The filariform larvae are then either expelled with stools or autoinfect, leading, in untreated patients, to a persistent infection that permits the survival of the *Strongyloides* for decades in the host and may contribute to the development of hyper infection syndrome [1,2].

Chronic strongyloidiasis is usually asymptomatic in immunocompetent hosts, but mild gastrointestinal, cutaneous and respiratory symptoms may occur. In addition, asymptomatic eosinophilia can be found in many chronically infected individuals [4,5].

However, in immunocompromised patients, the unresponsive immune system can allow a massive proliferation of the parasite, causing severe manifestations such as hyper infestation syndrome (HS) and disseminated strongyloidiasis (DS) [4,5]. HS is the process by which the parasite under- goes an uncontrolled replication within the host. Depending on the replication site, HS can lead to intestinal obstruction, perforation with gastrointestinal bleeding, pneumonitis, alveolar hemorrhage, respiratory failure or sepsis. DS happens when parasites spread to organs other than the usual replication sites (especially the liver, heart, kidneys and lymph nodes) [4,5].

Hematologic malignancies, transplant recipients, patients receiving high-dose corticosteroid therapy, HTLV-infected patients and patients suffering from alcohol abuse and malnutrition represent the most common risk factors for HS and DS [2].

To our knowledge, we have described the first European case of concomitant *Strongyloides* and Cytomegalovirus (CMV) infection in a patient affected by relapsed non-Hodgkin lymphoma and its diagnosis and management.

CMV reactivation in immunocompromised patients is a common event, considering that the adult population’s seroprevalence ranges between 60% and 100% [6]. In addition, an altered lymphocytic response may lead to viral replication and dissemination with specific organ diseases, mainly the gastrointestinal tract, the lungs and the retina [6,7,8].

The clinical manifestation of these two infections could overlap, affecting the same hematologic population and the same organs, often leading to a mis- or underdiagnosis.

## 2. Case Description

A 76-year-old woman affected by relapsed non-Hodgkin lymphoma was admitted for fever and diarrhea in October 2022. She had been diagnosed with B-cell non-Hodgkin lymphoma not otherwise specified (NOS) stage IV A in 2015 and achieved a first complete remission (CR) with standard R-CHOP (cyclophosphamide, doxorubicin, prednisone, rituximab and vincristine). She subsequently relapsed in 2019 with an indolent disease that required treatment in January 2022 with rituximab, polatuzumab and bendamustine and subsequently achieved a second metabolic CR. She further relapsed in September 2022 with advanced-stage lymphoma with kidney, pleural and bone marrow involvement causing pancytopenia and was started on third-line therapy with gemcitabine. The patient’s main comorbidities were: a previous diagnosis of breast cancer (in remission after surgery, chemotherapy and hormonal therapy); paroxysmal atrial fibrillation in anticoagulant treatment; and previous reactivation of CMV, treated with valgancyclovir.

**Figure 1:**
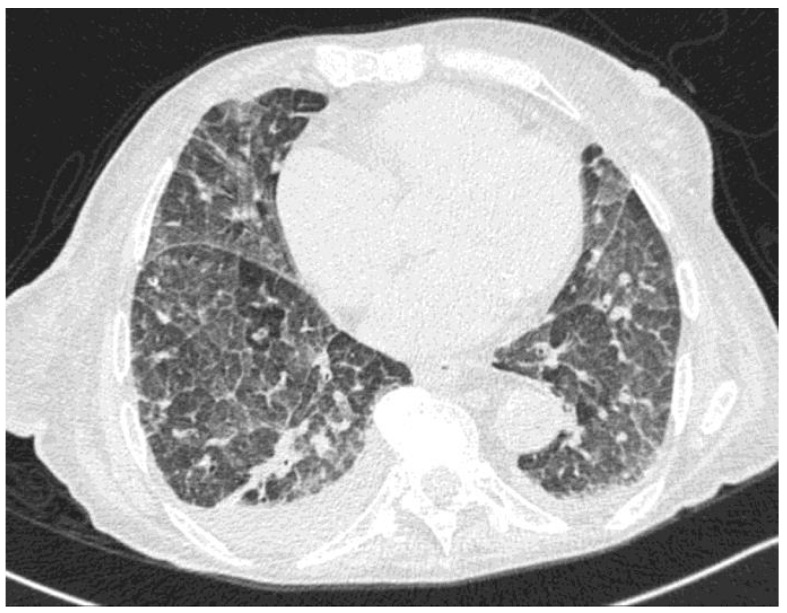
Chest CT showing diffuse ground-glass pattern and septal thickening.

At symptom presentation, the patient’s laboratory tests showed mild anemia and thrombocytopenia (White Blood Cell count 3670/µL, neutrophils 2890/µL, Hb 7.9 g/dL, platelets 95,000/µL), with normal renal and hepatic function. The C-reactive protein was 0.119 g/L. SARS-CoV-2 molecular testing was negative, and the chest X-ray did not show an acute lung involvement. She was started on ceftriaxone and levofloxacin, with an initial resolution of fever and a decrease of C-reactive protein (C-RP). However, after a few days, she developed acute respiratory dysfunction with hypercapnia. A chest CT (Figure 1) was performed and showed diffuse interstitial involvement with ground-glass opacity and no signs of pulmonary embolism.

Oxygen therapy and empirical treatment with trimethoprim/sulfametoxazole and ganciclovir were started, in the hypothesis of Pneumocystis jirovecii infection or CMV pneumonia. Moreover, the patient underwent a 4-day steroid regimen with methylprenisolone 40 mg every 12 h due to suspected Pneumocystis pneumonia. She was later transferred to the Intensive Care Unit due to the progressive worsening of respiratory insufficiency that required non-invasive ventilation and high-frequency atrial fibrillation. There, a large-spectrum antimicrobial therapy was started with meropenem and caspofungin, with no clinical improvement.

A beta-D-glucan and a film-array test on a nasal swab for adenovirus, coronavirus, metapneumovirus, rhinovirus, influenza A/B, parainfluenza and RSV both revealed negative results. CMV-DNA on blood was 21,964 cp/mL, and UL97 mutation for resistance to ganciclovir was negative.

The patient experienced a transient respiratory improvement with a decrease in CRP levels, and a bronchoalveolar lavage was performed (BAL).

**Figure 2:**
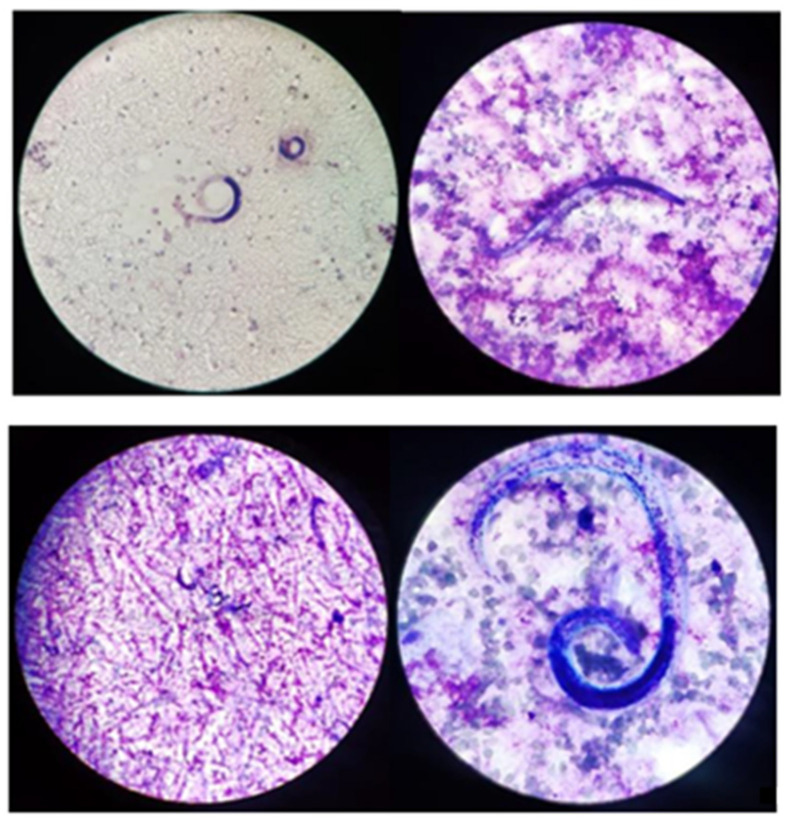
Microscopy observation of *S. stercoralis* on BAL (GIEMSA Coloration).

Forty-eight hours after bronchoalveolar lavage (BAL), samples turned positive for low count (10,000–100,000 CFU) *Enterococcus faecium* and *Candida krusei*. Moreover, CMV-DNA in the respiratory tract was up to 1,040,851 copies/mL and a high amount of *S. stercoralis* were found in a microscopic exam of BAL fluids (Figure 2).

Unfortunately, her clinical conditions rapidly worsened with respiratory and cardiac dysfunction and coma, and the patient died in the two hours after microbiological findings. Moreover, due to the rapid evolution of the disease, no other biological samples were obtained to assess the involvement of the gastro-intestinal system.

## 3. Discussion

To our knowledge, we have described the first case of a co-infection of *Strongyloides* and CMV in the Piedmont region (Italy) in an older woman with a relapse of non-Hodgkin lymphoma.

In over 43 years of analyzed literature, few immunosuppressed patients have reported concurrent *Strongyloides* spp. and CMV infection. As far as we know, no individuals with overlapping *Strongyloides* and CMV infections have been identified in a Euro- pean nation. *Strongyloides* epidemiology varies by nation and state, although CMV infection and reactivation risks are uniformly present worldwide. Most of the recorded cases took place in North and South America and Asia [2,3]. In their comprehensive analysis of the incidence of strongyloidiasis, Buonfrate and colleagues [2] rated the South-East Asian and American countries first and third in terms of the prevalence of *S. stercoralis* infection, with 6.9% (95% CI: 3.5–10.2%) and 12.1% (6.1–17.9%), respectively. Additionally, European nations showed a lower incidence following the same systematic review, with a rate of 2.8% (1.4–4.1%) [2].

In this report, we have described a case of an older woman in a lower-endemic country. In an interesting comparison between high and low endemic groups in Okinawa (Japan), Arakaki and colleagues [9] found that patients were older during *Strongyloides* diagnosis in an environment with lower incidence. Despite the low endemicity for *Strongyloides* in most areas of Northern Italy, we mention the work of Pirisi and colleagues [10] in which they presented a study regarding seroprevalence in elderly in-patients living in Novara (Piedmont), which is near the city in which our patient was born and lived for most of her life. Of interest, Pirisi et al. [10] also found serological solid evidence of an immune response to *S. stercoralis* in the elderly without eosinophilia at clinical presentation. Our patient presented eosinophils within normal values during all days of her hospital stay. Nevertheless, be- cause geoelmintiasis is neglected worldwide, seroprevalence is underestimated and we can hypothesize that data are lacking in a low-prevalence country.

In the cases described in the literature, a higher incidence of *Strongyloides*/CMV co-infection was described in patients who underwent solid organ transplantation, with a higher rate in kidney transplantation receivers [11,12,13,14,15,16,17,18,19,20]. In our search, we found only one patient with a lymphoma diagnosis and a concomitant *Strongyloides* and CMV co-infection [11]. Nonetheless, in the literature, different groups have summarized evidence of *Strongyloides* infection alone in lymphoma patients. For example, Genta and colleagues [21] reported 17 cases of *Strongyloides* infection in lymphoma patients, most of whom (N = 11) were identified after chemotherapy with (N = 9) or without (N = 2) steroids in treatment regimens. In subsequent work, Aydin and colleagues [22] reported three further cases. Of the now 20 patient cases identified, only one was in a European country (i.e., France).

There are several risk factors for developing *Strongyloides* hyperinflation/disseminated disease. In almost the totality of the cases described, a known cause of immune depression was reported [23]. Human T-lymphotropic virus type I (HTLV-1) [24], along with the administration of corticosteroids, are the main risk factors for the evolution to severe strongyloidiasis [25,26]. As previously reported, a higher rate of HS/DS has been described in patients who underwent solid or hematopoietic stem cell transplanta- tion and those who suffer from hematological and oncological malignancy for the disease and their specific treatment [27]. The patient underwent a 4-day steroid regimen with methylprednisolone 40 mg every 12 h due to suspected Pneumocystis jirovecii lung superinfection/reactivation.

A two- to three-fold increase in the risk of contracting *S. stercoralis* infection has been associated with corticosteroid therapy. Corticosteroids interfere with type-2 response binding the glucocorticoid receptors on the CD4+ Th2 cells, allowing a higher and faster proliferation of the parasites. Signs and symptoms of HS/DS have been reported to onset as early as a few days after the start of the treatment to as late as several months [25,28,29].

It is essential to highlight that our patient had previously undergone multiple courses of steroid treatment for lymphoma. It is still a mystery why the last course triggered the hyper infection and what mechanisms were responsible for changing the precarious balance between infection and immunologic infection control; from chronic uncomplicated autoinfection to uncontrolled fatal hyperinfection. It is possible that a combination of factors caused the hyperinfection. Gemcitabine, the last primary treatment for our patient, was reported in combination with cisplatin and steroids in at least two cases of HS with a urothelial and lung carcinoma, respectively [30,31].

Furthermore, we propose that the fast deterioration of the clinical manifestation of HS was related to the CMV-disseminated illness. Immunosuppression brought on by CMV infection is temporary but significant [32]. Furthermore, immunosuppression brought on by CMV in solid organ transplant recipients makes it easier for many pathogens to superinfect patients [33]. Notably, meta-analyses, including hundreds of transplant recipients, have demonstrated that anti-CMV prophylaxis protects against bacterial, fungal, and protozoan infections and fungi. [30].

If CMV prophylaxis is common in immunocompromised patients, *Strongyloides* prophylaxis in highly endemic areas has been proposed, especially in kidney transplantation recipients [20,25,33,34,35,36,37].

In several studies [25,34,35,36,37], using ivermectin as primary prophylaxis in high-endemic countries has shown some evidence of protection, and has been associated with a delayed occurrence of severe infection and reduced mortality compared to those who did not receive any prophylaxis. However, clinical trials have not assessed its benefit [20]. A retrospective cohort study conducted in Thailand did not demonstrate superior efficacy of empirical prophylaxis with ivermectin against strongyloidiasis in patients receving high-dose corticosteroids compared to a pre-emptive approach [38]. Miglioli-Galvao and colleagues, in a multicenter case-control study [20], failed to demonstrate the utility of albendazole prophylaxis in preventing the infection even if the delayed onset of the severe disease and reduction of mortality was observed (14% vs. 50%). The limit of this study may be found in the low efficacy of albendazole in the treatment of strogyloidiasis.

The first clinical manifestation in our patient was respiratory symptoms and radiological findings of interstitial pneumonia. After that, the most commonly reported symptoms in patients with *Strongyloides*/CMV infections involved the gastrointestinal system with colitis and peritonitis, followed by respiratory and skin manifestations. [11,12,13,14,15,16,17,18,19,20].

HS/DS and CMV disease usually singularly involve the gastrointestinal tract and lungs, causing colitis and interstitial pneumonia. The differential diagnosis is usually challenging due to the overlapping clinical presentations affecting the same population of immunocompromised patients. In high endemic areas, active surveillance for *Strongyloides* infection during CMV reactivation, and vice versa, should be mandatory.

Europe is considered a low incidence area for strongyloidiasis but autochthonous infections are still reported and sporadic trans- mission still occurs [39]. Northen Italy (Piedmont, Lombardy, Veneto and Friuli-Venezia-Giulia regions), along with the east coast of Spain and the south-west and north-east of France are the areas where the most cases of human strongyloidiasis have been reported [39]. These data are not enough to justify a systematic screening for patients at risk. A pre-emptive approach and increased clinical awarness in immunocompromised patients with exposure risks should be evaluated.

The low incidence, together with low awareness of the clinician, may lead to underdiagnoses of strongyloidiasis and contribute to maintaining its status as a neglected disease.

Due to the severity of the HS/DS-CMV co-infection and the delayed diagnosis and treatment, the mortality rate described in the literature is roughly above 50% despite therapy with ivermectin and ganciclovir. [11,12,13,14,15,16,17,18,19,20] A decline in the HS/DS mortality rate has been observed in recent decades, reaching a minimum of 28%, which is probably a result of increased monitoring [20]. De- spite this decline, the overall mortality is still unacceptably high. In kidney transplant recipients, mortality in donor-transmitted strongyloidiasis has been reported as high as 68% in HS/DS [16,40]. Respiratory failure and gram-negative bacteriemia have been indipendently associated with higher 30-day mortality [20]. In fact, HS/DS has been associated with Gram-negative bacteriemia that occurs in the translocation of the larvae form the intestinal tract as a knight-horse system [41]. In CMV-related colitis, this mechanism could be helped by the mucosal damage along with the immune impairment caused by the viral reactivation [32,41].

## 4. Conclusions

We presented the first European case of CMV/*Strongyloides* infections reported in literature to our knowledge.

With the information provided, we hope to increase awareness of this uncommon but lethal condition, especially in patients who have obvious risk factors and present symptoms consistent with HS/DS and CMV as well as rapid deterioration without alternative explanations. In locations with high seroprevalence, a *Strongyloides* preventive therapy might be helpful, although further research is required.

## Data Availability

Not applicable.
